# The Small RNA ncS35 Regulates Growth in *Burkholderia cenocepacia* J2315

**DOI:** 10.1128/mSphere.00579-17

**Published:** 2018-01-10

**Authors:** Sanne Kiekens, Andrea Sass, Filip Van Nieuwerburgh, Dieter Deforce, Tom Coenye

**Affiliations:** a Laboratory of Pharmaceutical Microbiology, Faculty of Pharmaceutical Sciences, Ghent University, Ghent, Belgium; b Laboratory of Pharmaceutical Biotechnology, Faculty of Pharmaceutical Sciences, Ghent University, Ghent, Belgium; University of Iowa

**Keywords:** *Burkholderia cenocepacia*, metabolism, small RNA

## Abstract

*Burkholderia cenocepacia* J2315 is a member of the *B. cepacia* complex. It has a large genome with three replicons and one plasmid; 7,261 genes code for annotated proteins, while 113 code for functional RNAs. Small regulatory RNAs of *B. cenocepacia* have not yet been functionally characterized. We investigated a small regulatory RNA, designated ncS35, that was discovered by differential RNA sequencing. Its expression under various conditions was quantified, and a deletion mutant, ΔncS35, was constructed. Compared to planktonic growth in a rich medium, the expression of ncS35 was elevated when *B. cenocepacia* J2315 was grown in biofilms and in minimal medium. Cells of the deletion mutant showed increased aggregation, higher metabolic activity, a higher growth rate, and an increased susceptibility to tobramycin. A transcriptomic analysis revealed upregulation of the phenylacetic acid and tryptophan degradation pathways in ΔncS35. Computational target prediction indicated that ncS35 likely interacts with the first gene of the tryptophan degradation pathway. Overall, we demonstrated that small RNA ncS35 is a noncoding RNA with an attenuating effect on the metabolic rate and growth. It is possible that slower growth protects *B. cenocepacia* J2315 against stressors acting on fast-dividing cells and enhances survival under unfavorable conditions.

**IMPORTANCE** Small RNAs play an important role in the survival of bacteria in diverse environments. We explored the physiological role of ncS35, a small RNA expressed in *B. cenocepacia* J2315, an opportunistic pathogen in cystic fibrosis patients. In cystic fibrosis patients, infections can lead to “cepacia syndrome,” a rapidly progressing and often fatal pneumonia. Infections with *Burkholderia* spp. are difficult to threat with antibiotics because of their high intrinsic resistance and ability to form biofilms. We show that ncS35 attenuates the growth and reduces the metabolic rate of *B. cenocepacia* and influences biofilm structure. This demonstrates that as-yet-uncharacterized small RNAs with regulatory function can influence physiological traits of *B. cenocepacia* that are relevant for infection.

## Abstract (precis)

Small RNAs play an important role in the survival of bacteria in diverse environments. We explored the physiological role of ncS35, a small RNA expressed in *B. cenocepacia* J2315, an opportunistic pathogen in cystic fibrosis patients. In cystic fibrosis patients, infections can lead to “cepacia syndrome,” a rapidly progressing and often fatal pneumonia. Infections with *Burkholderia* spp. are difficult to threat with antibiotics because of their high intrinsic resistance and ability to form biofilms. We show that ncS35 attenuates the growth and reduces the metabolic rate of *B. cenocepacia* and influences biofilm structure. This demonstrates that as-yet-uncharacterized small RNAs with regulatory function can influence physiological traits of *B. cenocepacia* that are relevant for infection.

## INTRODUCTION

*Burkholderia cenocepacia* is a member of the *B. cepacia* complex (Bcc). This group of bacteria consists of 20 closely related bacterial species found in different environments (1). Bcc bacteria can be isolated from soil or are associated with plants; they can be used as biocontrol or bioremediation agent or show plant growth-promoting activity (2). On the other hand, Bcc bacteria can also act as opportunistic pathogens, causing severe infections in immunocompromised patients (2). Especially individuals with cystic fibrosis (CF) or chronic granulomatous disease are sensitive to infections with Bcc bacteria. In CF patients, infections with *B. cenocepacia* can lead to “cepacia syndrome,” a rapidly progressing pneumonia, ultimately leading to death (3). Because of the high innate resistance to most antibiotics and because of their ability to form biofilms, eradication of Bcc bacteria is difficult (4, 5). *B. cenocepacia* isolate J2315 is a member of the highly transmissible ET12 lineage (3). It harbors two large replicons of 3.87 and 3.22 Mb, one smaller replicon of 0.88 Mb, and one plasmid of 0.09 Mb, in total encoding 7,261 annotated proteins (6). Genes on the largest replicon mainly encode core cellular functions, whereas the two other chromosomes mainly harbor genes that encode accessory functions (6). Besides protein coding genes, 113 RNA coding genes were annotated, including genes for 74 tRNAs and 10 riboswitches (6). Several studies have identified novel short transcripts in *B. cenocepacia* (7–10), but little is currently known about their function.

sRNAs are small noncoding RNA molecules in bacteria that possess regulatory functions on the posttranscriptional level (11). They are typically relatively short (40 to 500 nucleotides [nt]) and act mostly by base pairing with mRNA. *cis*-encoded sRNAs are located on the strand opposite their mRNA targets and are therefore fully complementary to their targets. *trans*-encoded sRNAs, the most abundant type of sRNAs, act by imperfect antisense base pairing on mRNA targets located elsewhere on the chromosome. Because of the limited complementarity to their targets, many *trans*-encoded sRNAs require the RNA chaperone protein Hfq to fulfill their function (12). By base pairing, the translation initiation frequency or stability of the mRNA target can be modulated. Binding of the sRNA within the 5′ untranslated region (UTR) can occlude the ribosome-binding site (RBS) and inhibit ribosome binding, leading to attenuation of translation. Untranslated mRNA can then be degraded by RNases. The translation initiation frequency can also be increased, as sRNAs can bind to hairpin structures of mRNA targets, open them, and reveal the RBS. Finally, mRNA stability can be directly affected by sRNAs, as binding within the coding sequence (CDS) of the mRNA target can result in increased degradation of the sRNA-mRNA duplex (13, 14).

sRNAs are typically only conserved between closely related species. They are involved in fast fine-tuning of gene expression, which is essential when bacteria have to survive stress, e.g., carbon or iron starvation, unfavorable pH or temperature, or oxidative and membrane stress. They are known to play a role in pathogenicity, by regulating the production of virulence factors, and in biofilm formation (14).

In a previous study, the transcriptome structure of *B. cenocepacia* J2315 was analyzed by differential RNA sequencing (dRNA-seq; 8, 15), which allows global mapping of transcription start sites (TSS) and discovery of novel transcripts. In the present study, the function of one short transcript discovered by dRNA-seq (8), designated ncS35, not associated with a coding sequence, and resulting in a short transcript, was further characterized.

## RESULTS

### Genome location and conservation of ncS35.

**FIG 1:**
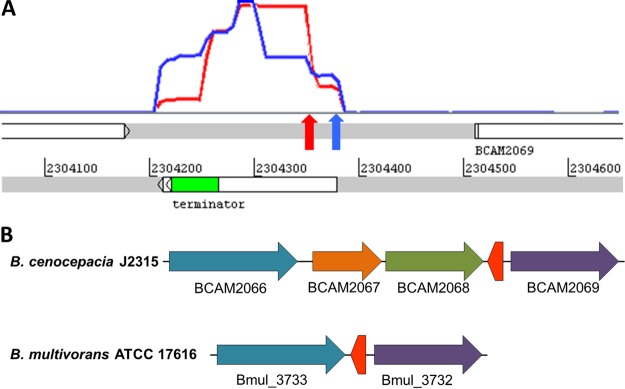
Genomic location of ncS35. (A) Coverage for ncS35 in dRNA-seq data. Blue line, TEX-treated subsample; red line, untreated subsample; blue arrow, location of TSS; red arrow, processing site. (B) Synteny of adjacent genes. The red arrow represents ncS35, located on the strand opposite its flanking genes, which encode a terpene cyclase (BCAM2068, green) and a conserved hypothetical protein (BCAM2069, purple). BCAM2067 (orange, a putative undecaprenyl pyrophosphate synthetase-encoding gene, *uppS*) and BCAM2068 are adjacent to ncS35 in *B. cenocepacia* strains J2315, K56-2, and H111 and in *B. dolosa* AU0158. In all of the other strains surveyed, the major facilitator protein (blue) is directly adjacent to ncS35 (*B. multivorans* ATCC 17616 is shown as a representative). Homologous genes have the same color code.

We previously identified an orphan TSS in an intergenic region on the second largest chromosome of *B. cenocepacia* J2315 (7, 8). This TSS is located on the opposite strand of its adjacent genes, BCAM2068 and BCAM2069 (Fig. 1), indicating an independently transcribed sRNA, which was designated ncS35. dRNA-seq data further indicated a processing site 29 nt downstream of the TSS, revealed by a coverage peak depleted in the Terminator RNA exonuclease (TEX)-treated subsample (Fig. 1A). 5′ rapid amplification of cDNA ends (RACE) confirmed the TSS as the beginning of the transcript (position 2304378), as well as the processing site (position 2304350). dRNA-seq data also showed an abrupt decrease in coverage at a distinct location (position 2304213), and 3′ RACE confirmed this as the end of the transcript.

The sequence of full-length ncS35 is conserved only within the Bcc, while the sequence corresponding to the processed form is also present in the *Burkholderia pseudomallei* group. Other bacterial lineages do not harbor ncS35. In Bcc species, the relative orientation of ncS35 is conserved but genes directly adjacent to ncS35 are not always homologous. In most of the strains investigated, ncS35 is flanked by a major facilitator superfamily protein and a conserved hypothetical protein (Fig. 1B). In *B. cenocepacia* strains J2315, K56-2, and H111 and in *Burkholderia dolosa* AU0158, two genes are inserted between ncS35 and the major facilitator superfamily protein (Fig. 1B).

Full-length ncS35 has a size of 166 nt and a computationally predicted secondary structure with four hairpins (see Fig. S1A in the supplemental material), a minimum free energy (MFE) of −77.80 kcal/mol, and a negative Z score for MFE of −3.97 (Fig. S1B). The processed form has a size of 138 nt, and its computationally predicted secondary structure, with an MFE of −67.10 kcal/mol and a Z score of −4.66, consists of three hairpins. The last hairpin, the rho-independent terminator, is followed by a stretch of five U residues.

10.1128/mSphere.00579-17.1 FIG S1  Secondary structure of ncS35. (A) Secondary structure of full-length ncS35 predicted by Mfold. The dotted line indicates the processing site. Orange circles indicate the reverse complement of the Northern blotting probe. Blue arrows indicate the annealing site of the 5′ ends of the forward and reverse primers used for qPCR and RACE. RACE primer ncS35-RB is the reverse complement of ncS35-FB. (B) MFE distribution of 500 randomly mononucleotide-shuffled sequences. The MFE of full-length ncS35 is −77.80 kcal/mol, and that of the processed form is −67.10 kcal/mol. Download FIG S1, DOCX file, 0.2 MB. Copyright © 2018 Kiekens et al. 2018 Kiekens et al. This content is distributed under the terms of the Creative Commons Attribution 4.0 International license.

### Expression of ncS35.

**FIG 2:**
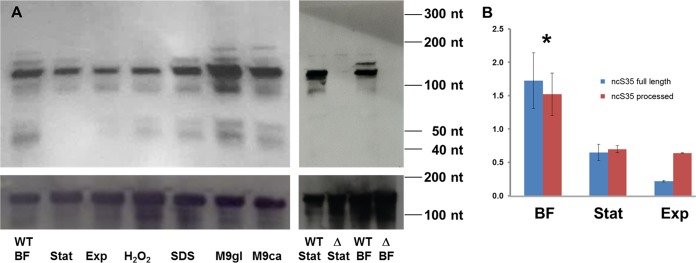
Expression of ncS35. (A) Northern blot assays. At the upper left is a Northern blot assay of ncS35 in wild-type (WT) *B. cenocepacia* J2315. Expression was evaluated in biofilms, stationary phase, and exponential phase. Responses to stress were evaluated in various media: LBB with 0.2% H_2_O_2_ added 15 min prior to harvesting, LBB supplemented with 0.005% (wt/vol) SDS (membrane stress), and M9 supplemented with either 10 mM glucose (M9gl) or 0.2% (wt/vol) Casamino Acids (M9ca; lower nutrient availability). 5S RNA was used as a loading control. At the upper right is a Northern blot assay of the wild type and ΔncS35 (Δ) that confirms the deletion of ncS35 and the specificity of probe hybridization. The lower parts of panel A are 5S rRNA loading controls. Full-size images of Northern blot assays are depicted in Fig. S6. (B) qPCR. Expression of ncS35 in *B. cenocepacia* J2315 was evaluated in exponential phase (Exp), stationary phase (Stat), and biofilms (BF) for full-length ncS35 (blue bars) and for both species combined (red bars). The locations of the primer pairs used are depicted in Fig. S1A. Fold changes were calculated relative to a cDNA standard (mixture of cDNA from all of the samples used in the experiment). Error bars represent standard deviations. ncS35 expression was significantly higher in biofilms than under all other conditions (*, *P* < 0.05; *n* = 3).

A search of the Rfam database did not result in any hits, so no indication of the function of ncS35 could be obtained by sequence comparison. To address that, ncS35 expression was tested under various growth and stress conditions. Northern blot assays showed that the expression of ncS35 was significantly higher in biofilms than in planktonic cultures (Fig. 2A). This was confirmed by quantitative reverse transcription-PCR (qPCR) with two different primer pairs detecting the full-length form or both forms, respectively (Fig. 2B; Fig. S1A). The presence of a dominant fragment with a size of approximately 140 nt and several less abundant fragments indicated that processed ncS35 is the most abundant form. Expression of ncS35 appeared to be increased in the presence of SDS and in minimal medium M9.

### Construction of ΔncS35.

To further investigate the function of ncS35, a deletion mutant, designated ΔncS35 was constructed. Whole-genome sequencing confirmed the deletion of ncS35 from the genome, as 165 nt (positions 2304238 to 2304402 on chromosome AM747721, spanning the entire locus of ncS35) were absent. Additionally, 15 nucleotide variants were detected in the deletion mutant (Data Set S1). Eight of these were associated with a transposase missing from a pseudogene within a genomic island, two were synonymous mutations, and three were nonsynonymous mutations: Thr708Ala in BCAL1675 (*amrB*), which encodes a multidrug efflux system transporter protein; Leu37Pro in BCAL3010 (*spoT*), which encodes a guanosine polyphosphate pyrophosphohydrolase; and Thr40Ile in BCAL3297, which encodes a ferritin-like DNA-binding protein. The mutation in BCAL3297 is located within the catalytic domain; the mutation in BCAL3010 is not within the catalytic domain. The two remaining nucleotide variants, located in intergenic regions, did not affect UTRs or promoter regions.

10.1128/mSphere.00579-17.7 DATA SET S1  Single nucleotide variants and indels in ΔncS35 compared to wild-type *B. cenocepacia* J2315 determined by whole-genome DNA sequencing. Download DATA SET S1, XLSX file, 0.02 MB. Copyright © 2018 Kiekens et al. 2018 Kiekens et al. This content is distributed under the terms of the Creative Commons Attribution 4.0 International license.

### Phenotype of ΔncS35.

The phenotype of the deletion mutant was investigated by using planktonic cultures and biofilms. Complementation experiments were performed with the wild type and ΔncS35 transformed with pM2 (vector control) and ΔncS35 transformed with pM2+ncS35, overexpressing full-size ncS35 from a rhamnose-inducible promoter (complemented ΔncS35).

**FIG 3:**
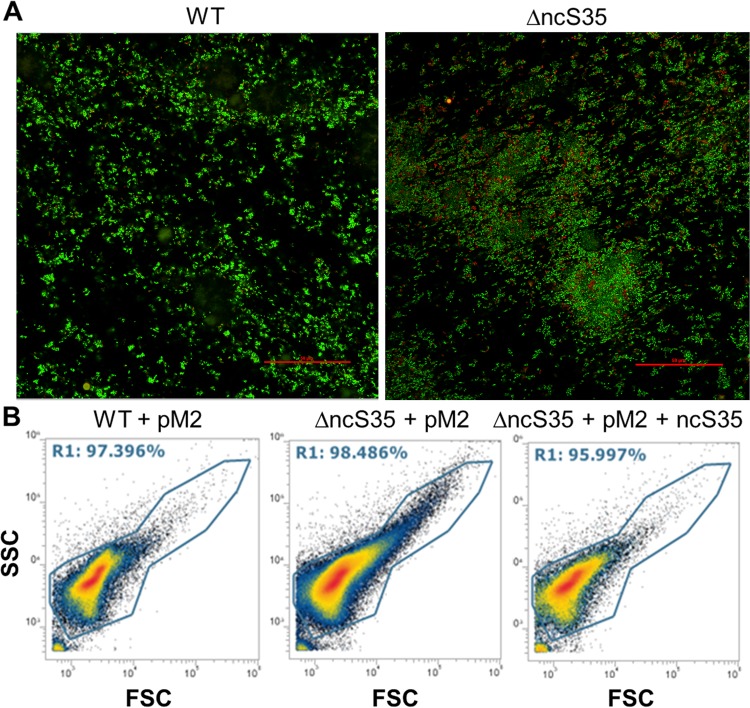
Cell aggregation in biofilms and planktonic culture. (A) Confocal laser scanning images. Shown are z-stack images of 24-h-old biofilms of the wild type (WT) and ΔncS35 after LIVE/DEAD staining. Scale bars, 50 µm. (B) Flow cytometry size/granularity plots of wild-type vector control (WT + pM2), ΔncS35 vector control (ΔncS35 + pM2), and complemented ΔncS35 (ΔncS35 + pM2 + ncS35) biofilm cells grown in LBB with 0.2% rhamnose and Tp at 600 µg/ml. The *x* axis represents forward scatter (FSC) and indicates cell size. The *y* axis represents side scatter (SSC) and shows cell granularity. Gate R10 represents all cells, and dots outside this gate are background fluorescence.

Confocal laser scanning microscopy revealed that ΔncS35 biofilm cells form larger aggregates than wild-type biofilm cells (Fig. 3A). However, no significant differences in biomass, metabolic activity, or the number of cultivable cells were observed (data not shown). When grown planktonically, ΔncS35 cells precipitated faster than wild-type cells (Fig. S2A). The wild-type phenotype was partially restored in the complemented mutant (Fig. S2B).

10.1128/mSphere.00579-17.2 FIG S2  Cell aggregation. (A) Sedimentation of planktonic cultures. O/N cultures of the wild type (WT) and ΔncS35 normalized to an OD of 1.0 in polycarbonate tubes were photographed over time. (B) Complementation of sedimentation. O/N cultures of the wild type vector control (WT + pM2), the ΔncS35 vector control (ΔncS35, + pM2), and complemented ΔncS35 (ΔncS35 + pM2 + ncS35) in LBB containing Tp at 600 µg/ml and 0.2% rhamnose. (C) Size/granularity plots of wild-type and ΔncS35 biofilm cells analyzed by flow cytometry. The *x* axis represents forward scatter (FSC) and indicates cell size. The *y* axis represents side scatter (SSC) and shows cell granularity. Gate R10 represents all of the cells; dots outside this gate are background fluorescence. Download FIG S2, DOCX file, 0.8 MB. Copyright © 2018 Kiekens et al. 2018 Kiekens et al. This content is distributed under the terms of the Creative Commons Attribution 4.0 International license.

Flow cytometry showed that ΔncS35 formed larger cellular aggregates with greater granularity in both planktonic cultures and biofilm (Fig. 3B; Fig. S2C). Aggregate formation of the complemented mutant was very similar to that of the wild type (Fig. 3B).

**FIG 4:**
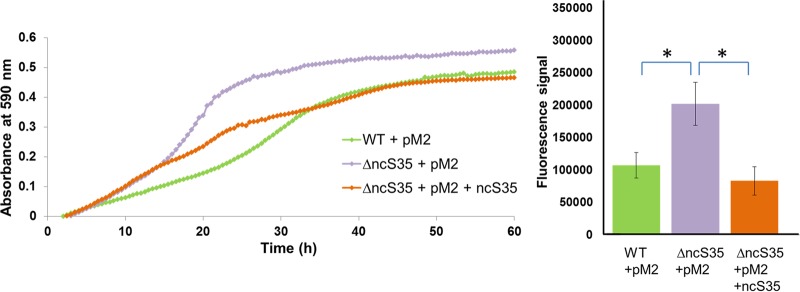
Complementation of growth and metabolic activity of planktonic cells. (A) Growth curve determined in LBB with Tp at 600 µg/ml and 0.2% rhamnose. Green line, wild-type vector control (WT + pM2); purple line, ΔncS35 vector control (ΔncS35 + pM2); orange line, complemented ΔncS35 (ΔncS35 + pM2 + ncS35). (B) Suspensions of wild-type vector control (WT + pM2), ΔncS35 vector control (ΔncS35 + pM2), and complemented ΔncS35 (ΔncS35 + pM2 + ncS35) cells were normalized to an OD of 1.0 and mixed with CellTiter-Blue, and fluorescence was measured after 1 h. Error bars represent standard deviations. Statistically significant differences are indicated by asterisks (*P* < 0.05; *n* = 3).

ΔncS35 grew to a higher optical density (OD) than the wild type (Fig. 4A; Fig. S3A); this phenotype could be complemented (Fig. 4A). The higher OD corresponded to a higher number of cultivable cells (Fig. S3D). During exponential growth, the slopes of the growth curves indicate that the ΔncS35 vector control and complemented ΔncS35 have a shorter doubling time (approximately 180 min) than the wild-type vector control (approximately 270 min). In later growth stages, the growth rate of the complemented mutant decreases to a greater extent than that of the mutant vector control. ΔncS35 colonies on agar plates appeared larger than wild-type colonies; also this phenotype could be complemented (Fig. S3B).

10.1128/mSphere.00579-17.3 FIG S3  Growth and metabolic activity of planktonic cells. (A) Representative growth curves of the wild type (WT) and ΔncS35 in LBB. The purple line is ΔncS35, and the green line is the wild type. (B) Dilution series of the wild type and ΔncS35 spotted on LBA and the wild type vector control (WT + pM2), the ΔncS35 vector control (ΔncS35, + pM2), and complemented ΔncS35 (ΔncS35 + pM2 + ncS35) spotted on LBB containing Tp at 600 µg/ml and 0.2% rhamnose. Cultures were normalized to an OD of 1.0 before dilution. Colonies were photographed after 24 h, and representative pictures are shown. (C) Suspensions of wild-type and ΔncS35 cells were mixed with CellTiter-Blue, and fluorescence was measured after 1 h. (D) CFU counts of the wild-type and ΔncS35 vector controls and complemented ΔncS35 grown in microtiter plates. Three samples (each sample is six wells pooled) per strain were plated. Samples were collected at the 40-h point on the growth curves (Fig. 4). Error bars represent standard deviations. Statistically significant differences are indicated by asterisks (*P* < 0.05; *n* = 3). Download FIG S3, DOCX file, 0.4 MB. Copyright © 2018 Kiekens et al. 2018 Kiekens et al. This content is distributed under the terms of the Creative Commons Attribution 4.0 International license.

Significantly higher metabolic activity (*P* < 0.05) was observed in planktonically grown ΔncS35 cells than in wild-type cells or cells of the complemented mutant (Fig. 4B; Fig. S3C).

ΔncS35 had the same tobramycin MIC (256 µg/ml) as the wild type. However, a lower OD of ΔncS35 was observed at concentrations near the MIC and partial complementation of this effect was possible (Fig. S4).

10.1128/mSphere.00579-17.4 FIG S4  Susceptibility of the wild type and ΔncS35 to tobramycin. ΔncS35 grows to a lower OD near the MIC, while the MIC is unchanged (left panel). The effect could be partially complemented (right panel). WT, wild type; pM2, empty-vector control; pM2+ncS35, vector containing ncS35. For complementation experiments, strains were grown in LBB containing Tp at 600 µg/ml and 0.2% rhamnose. After 24 h, the absorbance at 590 nm was measured. Representative graphs of four biological replicates are shown. Download FIG S4, DOCX file, 0.1 MB. Copyright © 2018 Kiekens et al. 2018 Kiekens et al. This content is distributed under the terms of the Creative Commons Attribution 4.0 International license.

### Differential gene expression in ΔncS35.

Gene expression in the wild type and ΔncS35 was quantified at different growth stages. In exponential phase, 364 genes were differentially regulated; in stationary phase, 386 were differentially regulated; and in biofilms, 1,676 were differentially regulated (Fig. S5 and Data Set S2). Fourteen genes were commonly upregulated in ΔncS35 compared to the wild type under all three conditions, and four genes were commonly downregulated.

10.1128/mSphere.00579-17.5 FIG S5  Gene expression of the wild type and Δnc35 under three growth conditions. The Venn diagrams show the numbers of genes up- and downregulated in Δnc35 compared to the wild type under planktonic exponential-phase, planktonic stationary-phase, and biofilm conditions. Only genes with significant differential expression (*P* < 0.05) and a change of >1.5-fold were included. Download FIG S5, DOCX file, 0.2 MB. Copyright © 2018 Kiekens et al. 2018 Kiekens et al. This content is distributed under the terms of the Creative Commons Attribution 4.0 International license.

10.1128/mSphere.00579-17.6 FIG S6  Full-size images of Northern blot assays. At the upper left is a Northern blot assay with probe ncS35-DIG and RNA extracted from wild-type (WT) cells grown under different conditions. The conditions, from left to right, are biofilm (BF), planktonic stationary phase (Stat), planktonic exponential phase (Exp), exponential phase in the presence of oxidative stress due to H_2_O_2_, exponential phase in the presence of 0.005% SDS, minimal medium with 10 mM glucose, and minimal medium with 0.2% (wt/vol) Casamino Acids. At the upper right is a loading control for the blot on the upper left. The membrane was stripped and reprobed with 5S RNA-digoxigenin. At the lower left is a Northern blot assay with probe ncS35-DIG and RNA extracted from stationary-phase planktonic cultures (Stat) and biofilms (BF) of the wild type and the ΔncS35 mutant (Δ). At the lower right is a loading control for the blot on the lower left. The membrane was stripped and reprobed with 5S RNA-digoxigenin. Download FIG S6, DOCX file, 1.1 MB. Copyright © 2018 Kiekens et al. 2018 Kiekens et al. This content is distributed under the terms of the Creative Commons Attribution 4.0 International license.

10.1128/mSphere.00579-17.8 DATA SET S2  Differential expression in ΔncS35 compared to wild-type *B. cenocepacia* J2315 determined by RNA-seq. Download DATA SET S2, XLSX file, 0.1 MB. Copyright © 2018 Kiekens et al. 2018 Kiekens et al. This content is distributed under the terms of the Creative Commons Attribution 4.0 International license.

**FIG 5:**
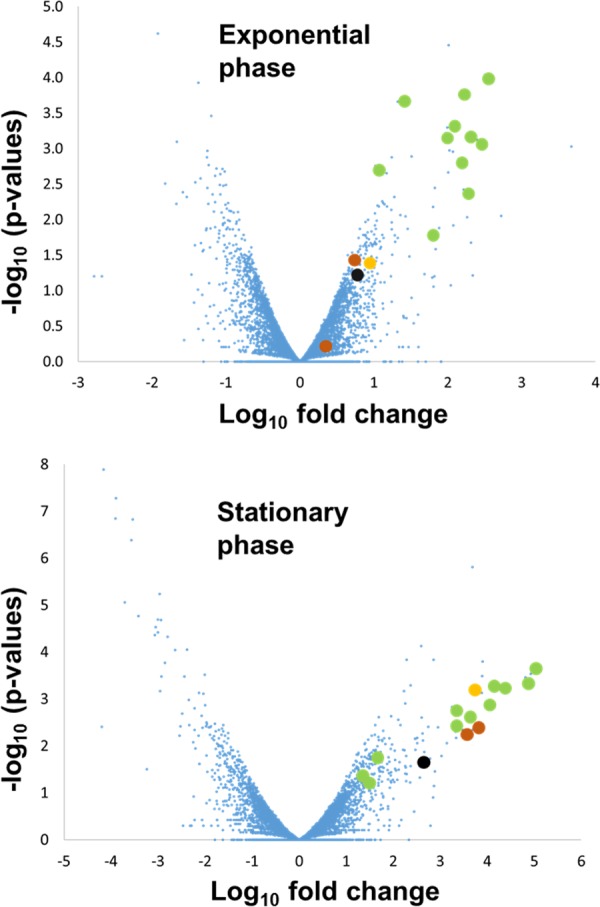
Volcano plot of gene expression data obtained by RNA sequencing. *x* axis: log_2_ of the fold difference between ΔncS35 and the wild type in the exponential and stationary phases. *y* axis: −log_10_ of the *P* value. Green dots represent genes involved in PAA degradation, and orange dots represent genes involved in tryptophan degradation. The black dot is BCAL0193, and the yellow dot is BCAL2790, two genes identified as putative targets of ncS35 by CopraRNA.

Selected gene expression changes are listed in Table 1. In exponential phase, genes involved in metabolic activity are differentially expressed, including genes that encode proteins for carbohydrate and amino acid uptake and metabolism, respiration, and ornibactin biosynthesis. Most upregulated in ΔncS35 is BCAM0166, which encodes a non-proton-pumping type II NADH dehydrogenase. Several genes for cable pilus biogenesis (BCAM2759, BCAM2762) are downregulated in ΔncS35. In stationary phase, as in exponential phase, genes involved in metabolic activity are differentially expressed. The most upregulated genes in stationary phase belong to the phenylacetic acid (PAA) degradation and tryptophan degradation pathways; these genes are also upregulated, but to a lesser extent, in exponential phase (Fig. 5). Genes involved in flagellar assembly (several loci) are also upregulated in stationary phase. Downregulated genes include those coding for the quorum sensing-regulated genes for zinc metalloprotease (*zmpA*, BCAS0409), adhesin protein (*bapA*, BCAM2143), and lectin (BCAM0184 to BCAM0186).

**TABLE 1:** Selected gene expression changes in ΔncS35 compared to the wild type

Function and gene	Exponential phase	Stationary phase	Biofilm	Annotation
PAA degradation pathway				
BCAL0212	3.8	19.9	–^a^	PAA degradation NADH oxidoreductase PaaE
BCAL0213	5.3	15.8	–	PAA degradation protein PaaD
BCAL0214	6.0	29.0	–	PAA degradation protein PaaC
BCAL0215	5.0	28.7	–	PAA degradation protein PaaB
BCAL0216	4.5	16.3	–	PAA degradation protein PaaA
BCAL0406	4.5	2.7	–	Probable enoyl-CoA^b^ hydratase PaaG
BCAL0407	5.1	5.1	–	β-Ketoadipyl-CoA thiolase
BCAL0408	4.5	9.0	1.9	PAA degradation oxidoreductase PaaZ
BCAM1711	5.4	12.5	–	Phenylacetate-coenzyme A ligase PaaK
BCAM1712	6.4	6.1	–	3-Hydroxybutyryl-CoA dehydrogenase
Tryptophan degradation pathway				
BCAL2790	2.0	14.0	–	Kynurenine formamidase
BCAL2791	1.9	10.6	–	Kynureninase
BCAL2792	–	12.3	−3.5	Tryptophan 2,3-dioxygenase
Amino acid transport and metabolism				
BCAL1055	2.6	2.1	1.7	Histidine transport system permease protein
BCAL1059	2.3	–	–	Succinylornithine transaminase
BCAL1060	2.6	–	–	Arginine *N*-succinyltransferase
BCAL1064	2.3	–	–	Succinylglutamate desuccinylase
BCAL2933	4.0	−4.0	−2.4	d-Amino acid dehydrogenase
Carbohydrate transport and metabolism				
BCAL1548	2.2	−3.3	−2.2	Sugar ABC transport system
BCAL1549	2.4	−3.0	–	Sugar ABC transport system
BCAL1550	2.3	–	–	Sugar ABC transport system
BCAL1661	2.0	–	–	Ribokinase
BCAL3038	2.0	–	–	ABC-type glycerol-3-phosphate transport
BCAL3039	2.0	–	–	ABC-type glycerol-3-phosphate transport
Ornibactin biosynthesis				
BCAL1696	2.3	–	–	Ornibactin biosynthesis protein
BCAL1697	2.8	–	–	Ornibactin biosynthesis protein
BCAL1698	2.5	–	–	Ornibactin biosynthesis protein
Respiration				
BCAL2141	–	13.4	3.5	Cytochrome *o* ubiquinol oxidase protein
BCAL2142	–	11.8	2.8	Cytochrome *o* ubiquinol oxidase subunit III
BCAL2143	–	13.2	2.5	Ubiquinol oxidase polypeptide I
BCAM2674	–	–	26.0	Cytochrome oxidase subunit I
BCAM0166	20.6	–	58.3	NADH dehydrogenase
BCAL3094	4.4	–	10.4	Oxygen-independent coproporphyrinogen III oxidase
BCAL2118	2.3	–	47.3	Isocitrate lyase
BCAM1588	–	–	11.5	Isocitrate lyase
Motility				
BCAL3506	–	5.1	–	Flagellar motor switch protein FliM
BCAL0140	–	4.6	–	Flagellar biosynthetic protein FlhB
BCAL0524	–	3.3	–	Flagellar motor switch protein FliG
BCAL0561	–	2.2	–	Flagellar synthesis protein FlgN
BCAL1677	–	3.4	–	Putative type 1 fimbrial protein
Surface protein				
BCAL3154	4.9	–	–	Glycine-rich surface protein
Pilus biogenesis				
BCAM2759	−2.0	–	−3.1	Putative minor pilin and initiator
BCAM2760	–	–	−6.5	Putative outer membrane usher
BCAM2761	–	–	−4.3	Giant cable pilus
BCAM2762	−2.8	–	−4.9	Giant cable pilus chaperone protein
BCAM2143	–	−3.0	−2.2	Cable pilus-associated adhesion protein
BCAL1528	–	−3.0	−3.2	Flp-type pilus assembly protein
BCAL1530	–	−3.0	−5.1	Flp-type pilus assembly protein
BCAL1531	–	–	−5.3	Flp-type pilus assembly protein
BCAL1532	–	–	−4.1	Flp-type pilus assembly protein
Quorum sensing-regulated genes				
BCAS0409	–	−5.4	−9.0	Zinc metalloprotease ZmpA
BCAM2143	–	−3.0	−2.2	Cable pilus-associated adhesin protein
BCAM0184	–	−6.4	−5.1	Lectin
BCAM0185	–	−4.7	−3.6	Lectin
BCAM0186	–	−6.1	−5.1	Lectin
Stress response				
BCAL1233	–	–	10.8	Heat shock protein Hsp20-related protein
BCAL1919	–	–	8.0	ClpB heat shock protein
BCAL2119	–	–	9.8	Universal stress protein family member
BCAL1234	–	2.6	12.0	Heat shock protein
Other genes, regulated under all three conditions				
BCAL0193	1.9	2.4	2.0	Exported protein
BCAL2206	−2.0	−4.8	−6.2	Phasin-like protein
BCAM1775	2.3	2.0	2.5	Transglycosylase-associated protein
BCAM2390	−1.9	−4.9	−5.2	Sarcosine oxidase delta subunit

a –, no significant >1.5-fold changes.

b CoA, coenzyme A.

Gene expression in ΔncS35 biofilms is characterized by downregulation of the expression of genes related to cell division, transcription, translation, and cable pilus biogenesis (BCAM2759 to BCAM2762). Genes with the highest upregulation include those that encode a low-oxygen-responsive regulator (BCAM0049), an alternative NADH dehydrogenase (BCAM0166), isocitrate lyase (BCAL2118), low-iron-responsive regulators FecI and FecR (BCAL1369 and BCAL1370), and certain chaperones and heat shock proteins.

### Computationally predicted targets of ncS35.

To predict putative direct targets of ncS35, we used CopraRNA, an algorithm that implements the accessibility of interaction sites and conservation of targets. Interactions were predicted for a stretch of 200 nt upstream to 100 nt downstream of the start codons of annotated genes and ranked according to combined *P* values, derived from all orthologous genes for which interactions were found. Of the consistently highest-ranking genes, 54 interactions were located within a UTR or a CDS and retained in full-length ncS35; 52 were found in the processed form, and 20 were found in both forms (Data Sets S3 and S4).

10.1128/mSphere.00579-17.9 DATA SET S3  Computationally predicted interactions for processed ncS35. Download DATA SET S3, XLSX file, 0.03 MB. Copyright © 2018 Kiekens et al. 2018 Kiekens et al. This content is distributed under the terms of the Creative Commons Attribution 4.0 International license.

10.1128/mSphere.00579-17.10 DATA SET S4  Computationally predicted interactions for full-length ncS35. Download DATA SET S4, XLSX file, 0.03 MB. Copyright © 2018 Kiekens et al. 2018 Kiekens et al. This content is distributed under the terms of the Creative Commons Attribution 4.0 International license.

The predicted target with the lowest energy score and highest statistical significance for both full-length and processed ncS35 is a gene that encodes an outer membrane protein (BCAM2255), with the predicted interaction site located inside the CDS, over a stretch of 40 nt. Other high-ranking predicted targets included genes that encode regulatory proteins, membrane transporters, lipoproteins, cytochrome *c* and other electron transport-related proteins, flagellar proteins, and ribosomal proteins. Most predicted interaction sites with *P* values of ≤0.01 were located inside the CDS of mRNA targets (Data Sets S3 and S4).

**FIG 6:**
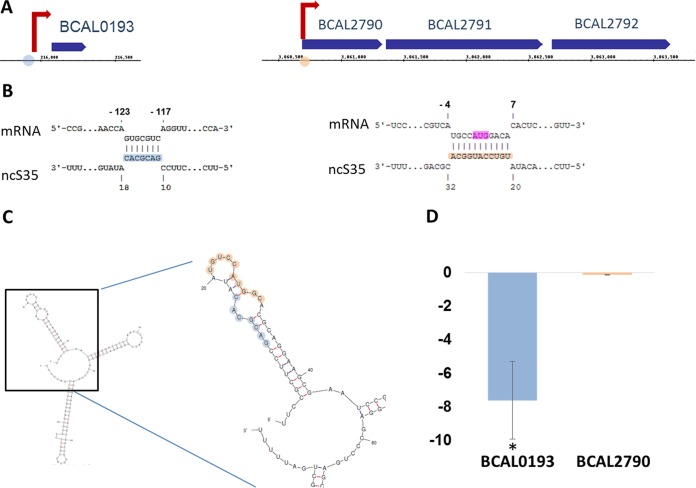
Locations of interaction sites for computationally predicted targets upregulated in ΔncS35. In blue is the interaction site for BCAL0193. In yellow is the interaction site for BCAL2790. (A) Locations of genes and TSS. Interaction sites of ncS35 on these targets are depicted as circles. (B) Interaction between ncS35 and mRNA. In pink is the start codon. (C) Secondary structure of processed ncS35 with computationally predicted interaction sites highlighted. (D) Fold change in expression when ncS35 is expressed in *trans* in the ΔncS35 mutant in the exponential phase. BCAL0193 (left) is downregulated, and BCAL2790 (right) expression does not change. A statistically significant difference is indicated by the asterisk (*P* < 0.05; *n* = 3).

Only two genes on the list of highest-ranking predicted targets for the processed sRNA have altered expression in ΔncS35 (Fig. 6). BCAL0193, which encodes an exported protein with an unknown function is upregulated in the mutant under all three conditions. The second gene (BCAL2790) encodes an arylformamidase and is the first gene in an operon containing genes for tryptophan degradation; it is upregulated in the mutant in the exponential and stationary phases. In both cases, the predicted interaction is directly adjacent to the TSS of the genes (Fig. 6A); in the case of BCAL2790, the interaction site includes the start codon (Fig. 6B). Upregulation of BCAL0193 could be complemented by ncS35 expression in *trans* (Fig. 6D), while expression of BCAL2790 was not affected by complementation. The predicted target with the lowest energy score and the highest statistical significance (BCAM2255) did not change expression significantly.

## DISCUSSION

In the present study, ncS35, a novel sRNA of *B. cenocepacia* J2315, is described. Its predicted secondary structure is thermodynamically favorable, with stable stem-loops and a negative Z score for MFE. Known regulatory sRNAs of other bacterial species tend to have a low Z score, which serves as an indicator of their structural significance (16), as it confirms that the actual RNA sequence has a significantly lower MFE than sequences with the same length and nucleotide composition. The negative Z score of ncS35 therefore indicates that ncS35 has a function in *B. cenocepacia* J2315. The processed form of ncS35 is more abundant and conserved in more distantly related *Burkholderia* species than full-length ncS35 is; this suggests that the processed form is the functional RNA species.

Construction of an ncS35 deletion mutant was possible, and its viability was not affected, showing that ncS35 is nonessential under laboratory conditions. However, the mutant had additional mutations, notably, nonsilent point mutations in three genes. The method used for mutant construction (17) includes repeated selection steps where single colonies are further propagated after having grown for 3 days on agar containing large amounts of antibiotics. Mutations could have arisen at random but could also have been selected for by the growth conditions. The mutation in an efflux pump points toward selection by antibiotics. The mutation in *spoT* could reduce its guanosine pentaphosphate (ppGpp)-hydrolyzing activity, resulting in higher levels of ppGpp, which is beneficial for survival in the presence of trimethoprim (Tp) (18). On the other hand, a mutation in *spoT* could also represent a suppressor mutation, partially counteracting the growth rate-increasing effect of ncS35 deletion.

Because of the additional mutations, complementation by overexpression of ncS35 in *trans* in the mutant was required to link phenotypic changes to the deletion of ncS35. All major phenotypic changes could be complemented, i.e., the attenuating effect of ncS35 on growth, metabolic activity, cell aggregation, and susceptibility to tobramycin.

sRNAs that attenuate growth have been described in *Escherichia coli* (Spot 42) (19) and *Bacillus subtilis* (RnaC) (20). Expression of Spot 42 is negatively regulated by cAMP and increases in media containing glucose. Spot 42 has a role in central carbon metabolism, and mutants overexpressing Spot 42 show a small-colony phenotype (21). RnaC is involved in regulation of the growth rate and entry into stationary phase in *B. subtilis*. The impact of ncS35 on the growth of *B. cenocepacia* could be similar to that of Spot 42 and RnaC, despite the lack of sequence similarity. The increased expression of ncS35 in minimal medium, particularly in the presence of glucose, suggests a possible role for cAMP in its regulation. sRNAs that affect aggregate formation also have been described (22), albeit in most cases as positive regulators of aggregate formation, in contrast to the negative regulation observed in the present study.

The lower OD of the mutant observed at tobramycin levels near the MIC could be an indirect effect of the higher growth rate and higher metabolic activity of ΔncS35. Faster growing cells could be more susceptible to antibiotics, in particular to those that inhibit translation, such as tobramycin (23, 24). Moreover, uptake of aminoglycosides is proton motive force dependent (25) and could therefore be stimulated by a higher respiration rate.

To identify the mechanism by which ncS35 attenuates growth and causes cell aggregation, RNA-seq and computational target prediction were performed with the aim of finding differentially expressed genes that also have a high probability of being directly targeted by ncS35. sRNA binding can directly affect mRNA stability, as sRNA-mRNA duplexes can trigger cleaving by RNases or occlude a cleaving site (26). Binding to a 5′ UTR can affect translation initiation, which can indirectly affect mRNA degradation via changes in ribosome binding to mRNA. Ribosome binding provides protection against RNases, and hence, when binding is attenuated, the mRNA degradation rate increases (26), and these changes can be detected by RNA-seq. Most experimentally confirmed interactions between sRNAs and mRNA lead to repression of gene expression (26), resulting in upregulation of target gene expression in a deletion mutant.

Among the highest ranking computationally predicted interactions, only two genes change expression on the mRNA level; both are upregulated. The predicted interaction sites for BCAL0193 and BCAL2790 are located within the 5′ UTR of the respective genes and near their TSS, as determined by dRNA-seq (8). The interaction site for BCAL2790 overlaps the start codon, while the interaction site for BCAL0193 overlaps a potential alternative start codon, GTG, that is in frame with the annotated gene. In both cases, it is possible that sRNA binding affects translation. Changes in mRNA abundance would therefore be caused indirectly by increased degradation of the untranslated mRNA. Only upregulation of BCAL0193 could be complemented when ncS35 was expressed in *trans*. One reason for that could be that upregulation of BCAL2790 is not directly linked to ncS35 and the presence of this gene among the highest ranking predicted targets is due to a false-positive prediction. Another possible reason is that the conditions under which ncS35 exerts its effect on BCAL2790 were not met in the complementation experiment. The effect of ncS35 is growth stage dependent, exemplified by the little overlap in genes changing expression in the mutant under the three conditions. During complementation, Tp and rhamnose were added to the medium, and BCAL2790 was not expressed in stationary phase in the mutant, in contrast to the RNA-seq experiment.

Either computationally predicted targets not changing expression in our analysis are false positives, or the conditions and method of analysis selected were not suitable to detect any interaction. Targeted genes could change expression solely on the posttranscriptional level, or proteins could be targeted directly without a requirement for interaction with mRNA (27). On the other hand, the strategy selected for computational prediction could be unsuitable to point out direct targets of ncS35, if true interactions were not among the highest ranking ones.

Several gene expression changes observed for the deletion mutant in planktonic exponential- and stationary-phase cultures point toward greater metabolic flux, which can account for the higher growth rate, as well as for the higher metabolic activity. The uptake and metabolism of numerous organic compounds are upregulated. The increased expression of tryptophan degradation could indirectly cause the upregulation of the PAA degradation (PAA) pathway in the exponential and stationary phases. These genes are among those with the highest fold upregulation in the mutant. An aromatic compound that is assumed to induce the PAA degradation pathway is hydroxyanthranilic acid, an intermediate in the degradation of tryptophan in *Burkholderia* spp. (28, 29). Tryptophan degradation and PAA degradation could therefore be linked. Upregulation of PAA degradation can also occur by a decrease in PaaR expression, a decrease in the intracellular glucose concentration, or an increase in the levels of aromatic precursors (29, 30), but no indication was found that ncS35 directly regulates PaaR or glucose metabolism. Interestingly, in *E. coli*, a confirmed target for Spot 42 is *paaK* (19), which is involved in PAA degradation. However, direct targeting of these pathways by ncS35 could not be confirmed.

The higher metabolic flux can, in turn, indirectly cause the upregulation of non-proton-translocating type II NADH dehydrogenase and the glyoxylate shunt. The use of the alternative NADH dehydrogenase can contribute to the maintenance of NAD^+^/NADH balance at high metabolic rates (31). Additionally, the use of a non-proton-translocating NADH dehydrogenase and the glyoxylate shunt can reduce the production of reactive oxygen species during aerobic growth and thus protect the cell from damage (31, 32).

Higher values for ΔncS35 in the CellTiter-Blue assay indicate that levels of NADH are increased in the mutant. This assay measures the reduction of resazurin to fluorescent resofurin by NADH inside the cytoplasm. It is usually used as a proxy test to determine the number of viable cells in, e.g., a biofilm (33). However, in this case, the cell numbers at the point of measurement were normalized to a fixed OD. The increased reduction of resazurin in the mutant cell suspension is therefore more likely to be due to the increased availability of NADH for resazurin reduction than to higher cell numbers. Reduction of the NADH levels in the cell by using type II NADH dehydrogenase and the glyoxylate shunt could be beneficial for the maintenance of metabolic flux.

The increased aggregation of ΔncS35 observed could be linked to several changes in gene expression, i.e., upregulation of flagellar genes (several loci), a fimbrial protein gene (BCAL1677), and/or a glycine-rich surface protein gene (BCAL3154). Changes in the expression of genes involved in cable pilus biogenesis (downregulated in exponential phase and in biofilms) could also be responsible for this increased aggregation, as in a study on *B. cenocepacia* J2315 mutants disrupted in cable pilus biogenesis, more autoaggregation and a higher precipitation rate than for wild-type cells were observed (34).

## MATERIALS AND METHODS

### Bacterial strains, plasmids, and culture conditions.

The bacterial strains and plasmids used in this study are listed in Table 2. Bacteria were maintained on Luria-Bertani agar (LBA; Oxoid) at 37°C, and liquid overnight (O/N) cultures were grown in Luria-Bertani broth (LBB; Oxoid) at 37°C with orbital agitation (150 rpm). Where appropriate, the following antibiotics were added for plasmid selection: ampicillin (Sigma-Aldrich), chloramphenicol (Sigma-Aldrich), gentamicin (Sigma-Aldrich), kanamycin (Sigma-Aldrich), Tp (Ludeco), and tetracycline (Sigma-Aldrich). Overexpression mutants were grown in LBB supplemented with Tp at 600 µg/ml and 0.2% (wt/vol) rhamnose. M9 medium was used as a minimal medium. *B. cenocepacia* J2315 is auxotrophic for phenylalanine; therefore, in the absence of a source of amino acids, 0.5 mM phenylalanine was added.

**TABLE 2:** Bacterial strains and plasmids used in this study

Strain or plasmid	Description	Reference or source
*B. cenocepacia*		
J2315 (LMG 16656)	CF sputum isolate	BCCM/LMG collection
ΔncS35	sRNA ncS35 deletion mutant	This study
*E. coli*		
DH5α	Maintenance of replicative plasmids	Lab stock
DH5α λ*pir*	Maintenance of suicide plasmids with *ori*_R6K_	Biomedal, Seville, Spain
JM109	Cloning of PCR products	Promega, Leiden, The Netherlands
Plasmids		
pGEM	Parental vector for cloning of PCR products, *ori*_pUC_, *ori*_F1_, Amp^r^	Promega
pRK2013	Helper plasmid, *ori*_colEI_, Km^r^	46
pGPI-SceI-XCm	Suicide plasmid, *ori*_R6K_, I-SceI restriction site, Tp^r^ Cm^r^	38
pDAI-SceI-SacB	Broad-host-range replicative plasmid, *ori*_*pBBR1*_, I-SceI nuclease, counterselectable marker SacB, Tet^r^	38
pSCrhaB2	Expression vector containing a rhamnose-inducible promoter, *ori*_*pBBR1*_, *rhaR*, *rhaS-P*_*rhaB*_, Tp^r^	39
pM2	pSCrhaB2 lacking Shine-Dalgarno sequence and start codon	This study
pM2+ncS35	pSCrhaM2 overexpressing sRNA ncS35	This study

Planktonic cultures were grown at 37°C with orbital agitation (150 rpm). For the exponential phase, cells were harvested at an OD of 0.5 (5 × 10^8^ CFU/ml); for the stationary phase, cells were harvested at an OD of 2.0 (2 × 10^9^ CFU/ml). Biofilms were cultivated in microtiter plates (35). To harvest biofilm cells, biofilms grown for 24 h were rinsed with physiological saline (PS). To detach the cells, 100 µl of PS was added to each well and the plate was sonicated at 40 kHz and shaken at 900 rpm for 5 min. Cells from two cycles of shaking and sonication were pooled and collected in one tube.

### Biofilm analysis.

Biomass was quantified by using a crystal violet assay, CFU counts were determined by plating, and cell viability was determined by a resazurin-based assay as described by Peeters et al. (33). For confocal laser scanning microscopy, biofilms were grown in a 96-well plate with a glass bottom (Greiner Bio-One), stained with a LIVE/DEAD solution (0.3% SYTO 9 and propidium iodide in PS; Life Technologies) for 15 min, and visualized with a motorized Nikon TE2000-E inverted microscope (Nikon Benelux) (36).

### Growth and metabolic activity.

Growth curves were measured in LBB medium. A 5 × 10^5^-CFU/ml inoculum was added at 200 µl/well to a round-bottom 96-well plate, and the absorbance at 590 nm was measured for 60 h in a microplate reader (Envision; PerkinElmer). CFU counts were determined by plating. Cells were grown as described for growth curves, and triplicate samples were taken after 40 h of incubation. For every sample, the content of six wells was pooled in a microcentrifuge tube and subjected to two rounds of sonication for 5 min, followed by vortexing for 1 min. Duplicate dilution series of each sample were prepared. To assess the sedimentation rate, planktonic cultures normalized to an OD of 1.0 were left undisturbed and representative pictures were taken. To measure metabolic activity, planktonic cultures normalized to an OD of 1.0 were centrifuged and resuspended in 100 µl of PS with 20 µl of CellTiter-Blue (Promega) solution. After incubation for 1 h at 37°C, fluorescence was determined (excitation wavelength, 560 nm; emission wavelength, 590 nm).

### Determination of susceptibility to tobramycin.

The MIC of tobramycin (TCI Europe) was determined in accordance with EUCAST guidelines by using flat-bottom 96-well plates. Growth was evaluated after 24 and 48 h of incubation at 37°C by measuring absorbance at 590 nm. The MIC was the lowest concentration at which no difference in absorbance from that of uninoculated medium was measured.

### Flow cytometry.

Cell size and granularity of planktonic cultures and biofilms were measured with an Attune Nxt flow cytometer and autosampler from Invitrogen with the Attune Nxt software, version 5.2 (Thermo Fisher). Planktonic cultures were investigated by using a suspension with an OD of 0.1 (1 × 10^7^ CFU/ml). For biofilms, cells were grown and detached as described before. All media and solutions were filter sterilized before use (Puradisc FP30; Whatman). Cells were collected in a round-bottom 96-well plate and quantified with excitation at 488 nm with a blue laser and appropriate filters.

### DNA extraction.

Genomic DNA was isolated after mechanical disruption with glass beads by a modified bead-beater protocol (37).

### Construction of mutants.

A deletion mutant, ΔncS35, was constructed by allelic recombination (17, 38). The primers used for amplification of flanking regions are listed in Table 3. Correct insertions were confirmed by Sanger sequencing.

**TABLE 3:** Primers and probes used in this study

Purpose and primer or probe	Oligonucleotide sequence (5′–3′)^a^
Construction of deletion mutant	
Upstream flanking sequence	
UM2068-F	TAT**GAATTC**TGATCGCCGACCCGAC
UM2068-R	AAA**GCTAGC**CCGTGTGGATGTTCGC
Downstream flanking sequence	
DM2069-F	TAA**GCTAGC**CGAAACGCATCTGTCAACC
DM2069-R	TAT**AGATCT**CCAGTCGTCGATCTGCACC
Confirmation of deletion	
ncS35join-F	CACATACATTCGCGGCAACT
ncS35join-R	CGAGCATCTTGTAGCGCATC
Construction of modified plasmid pSCrhaM2	
M2-F	TTACTAGTAAGGTACCCGGGGATCCTCTAGAGT
M2-R	ATTACGACCAGTCTAAAAAGCGCCTG
Construction of overexpression mutant	
ncS35ov-F	AT**ACTAGT**ATGGCGCGACGACAAGTG
ncS35ov-R	TTT**AAGCTT**GCCGCGACATGACCTGT
Northern blotting probes	
ncS35-DIG	DIG-TTGAGAGTCCCGGATTC
5S RNA-DIG	DIG-AGAGTCGTTTCACGGTC
RACE and qPCR	
ncS35-FA	GACAAGTGCGCGCAACGA
ncS35-FB	ACATATGTCCATGGCACGCAG
ncS35-RB	CTGCGTGCCATGGACATATGT
ncS35-RC	TGGATGTTCGCTCAGGGCTC
L0193F	GAAGACGCTCGCTTCGATCA
L0193R	TCGGCTTGCTGTGATCCTTC
L2790F	CGACTCACGCTTCGTCATGC
L2790R	GGCCTCCATCCGCCATACG
Control genes	
BCAM0918F RpoD	GAGATGAGCACCGATCACAC
BCAM0918R	CCTTCGAGGAACGACTTCAG
BCAL0026F ParA	TATGAAGTGCTGGTCGATGG
BCAL0026R	TCAGCACGAAATCGTAGTCG
BCAL0813F RpoN	AGCTCAATCCGGAAGTCGTG
BCAL0813R	AGCTGCTGTTTCAGCGATCC
BCAL2367F	ACCATTTCCGCAACAAGGAC
BCAL2367R	TGAAATCGGCCATGTACTGC
BCAL0972F	TCTCGAAGGTCTGGCACGAG
BCAL0972R	CGTGATGTCGTGCTTCATCG
BCAL1895F SurE	CAGCGGGTACGGGTTTCTTC
BCAL1895R	GTTCTGGCCGTTGTTGATGC
BCAL2553F	TGATCTGGGTGGTCAAGCTG
BCAL2553R	TGCAGGTCAAAATCGTCGTC
BCAS0059F TraD	ATGCGGAATTCCAACAGGAG
BCAS0059R	GCCCTTGCTCGAATAGTTGG

a DIG, digoxigenin. Restriction sites are in bold type.

For complementation experiments, an RNA expression plasmid, pSCrhaM2, was constructed from pSCrhaB2 (39) by removing its Shine-Dalgarno sequence and start codon by inverse PCR with M2 primers (Table 3). DNA was amplified with the LongRange PCR kit (Qiagen) with plasmid pSCrhaB2 as the template. The PCR product was blunted with T4 DNA polymerase and self-ligated. The absence of the Shine-Dalgarno sequence and start codon, as well as the integrity of the multiple cloning site, was confirmed by Sanger sequencing. sRNA ncS35 was cloned into pSCrhaM2 with ncS35ov primers (Table 3). Plasmids were transformed into ΔncS35 by triparental mating. The wild-type and ΔncS35 strains transformed with empty pSCrhaM2 were used as a vector control. All of the media used for mutant propagation and experiments contained Tp at 600 µg/ml. Rhamnose was added, when required, to a final concentration of 0.2% (wt/vol).

### DNA sequencing.

Both the wild type and ΔncS35 were sequenced. An Illumina paired-end library was generated from 1 µg of genomic DNA. The DNA was fragmented to 200 bp by Covaris S2 sonication (duty cycle, 10%; intensity, 5; number of cycles per burst, 200; treatment time, 180 s), and a sequence library was made for each sample with the TruSeq DNA PCR free library preparation kit (Illumina). The library was sequenced on an Illumina NextSeq 500, generating 75-bp single reads.

Sequencing reads from the wild type and ΔncS35 were mapped to the *B. cenocepacia* J2315 reference sequence (6) with CLC Genomics Workbench by using a 50% length fraction cutoff and an 80% similarity cutoff. The CLC Genomics Workbench Basic Variant Detection tool was used to detect single nucleotide polymorphisms, and the Indels and Structural Variants tool was used to detect genome insertions and deletions. Variations of the reference sequence that were present in more than half of the reads in ΔncS35 but not in the wild type were reported.

### RNA extraction.

RNA was extracted as described by Sass et al. (7).

### RACE.

5′ RACE and 3′ RACE with biofilm RNA were performed as described by Sass et al. (7). The gene-specific primers used are listed in Table 3.

### qPCR analysis.

Total RNA was treated with DNase a second time for 60 min, purified with phenol-chloroform (Roti-Aqua-P/C/I; Carl Roth), and precipitated O/N at −20°C with ethanol-sodium acetate (30:1 ratio of ethanol to 3 M sodium acetate, pH 6.5). After centrifugation and washing with 70% ethanol, the RNA pellet was air dried and redissolved in water.

qPCR of three biological replicates was performed as described by Sass et al. (7). The primer pairs used were ncS35-FA with ncS35-RC (detecting full-length ncS35) and ncS35-FB with ncS35-RC (detecting both forms) (Table 3; Fig. S1A). Data were normalized to eight control genes, moderately expressed genes that did not change expression in a microarray reference data set (Table 3) (40). The final fold changes were calculated relative to a cDNA standard, a mixture of cDNA of all of the samples under all of the conditions tested. Normality of distribution was tested with a Kolmogorov-Smirnov test, and changes in expression were analyzed by one-way analysis of variance (ANOVA) with a Tukey *post hoc* test by using SPSS, Inc., Statistics v. 24 (IBM) on log-transformed fold changes.

### Northern blot assays.

Northern blotting was performed as described by Sass et al. (7). Three biological replicates were blotted, and representative images are shown.

### RNA sequencing and data analysis.

For each condition, three biological replicates were sequenced. RNA concentrations were measured with the Quant-iT RiboGreen RNA assay (Life Technologies, Inc.), and RNA quality was assessed by capillary electrophoresis with the RNA 6000 Pico Chip (Agilent Technologies). A 2-µg sample of RNA was depleted of rRNA with the Ribo-Zero rRNA Removal kit for Gram-negative bacteria (Illumina). Library preparation was performed in accordance with the TruSeq Stranded Total RNA Library Preparation protocol (Illumina). Libraries were quantified by qPCR in accordance with the Illumina Sequencing Library qPCR Quantification protocol guide, version February 2011. Library size distribution and quality were checked on a DNA 1000 chip (Agilent Technologies). Sequencing was performed with a high-throughput Illumina NextSeq 500 flow cell generating 75-bp single reads.

Reads were mapped to the *B. cenocepacia* LMG 16656 reference genome (6) with a 95% similarity cutoff by using CLC Genomics Workbench version 8.5.1 (Qiagen). Statistical analyses were performed with EDGE (Estimated Degree of Gene Expression). Genes were reported as significantly differentially expressed when the *P* value was <0.05 and there was a change of ≥1.5-fold.

### Computational sequence analyses.

BLASTn (41) was used to search for sRNA homologues with the following input parameters: a word size of 7, match/mismatch scores of 1/−1, a gap existence cost of −0, and a gap extension cost of 2. The cutoff was 65% for query coverage and 60% for sequence similarity. Thirteen Bcc strains whose genomes have been sequenced and nine non-Bcc strains were included in the BLASTn search. ncS35 was compared to the Rfam database (42) to find homologues with known functions. The Mfold web server was used to predict secondary structure by using standard parameters (43). Z scores for MFE were calculated by using 500 randomly mononucleotide-shuffled sequences (CLC Genomics Workbench v. 8.5.1). The Z score is the number of standard deviations (σ) by which the MFE of the actual sequence (*x*) deviates from the mean MFE (μ) of shuffled sequences, i.e., (*x* − μ)/σ.

### Computational target prediction.

Putative targets of ncS35 were predicted with CopraRNA (Comparative Prediction Algorithm for sRNA Targets) (44). The underlying IntaRNA parameters for implementation of the CopraRNA algorithm are a seed length of 7, a target folding window size of 150, and a maximum base pair distance of 100 (45). Interactions were predicted for a window of 200 nt upstream to 100 nt downstream of the start codon because most experimentally verified sRNA-target interactions are found in this region (44). Eight Bcc strains whose genomes have been sequenced were used as the input (*B. cenocepacia* J2315, AU1054, MC0-3, and HI2424; *B. lata* 383; *B. vietnamiensis* G4; *B. multivorans* ATCC 17616; and *B. ambifaria* AMMD). Using rather closely related strains has the advantage of including all of the essential and otherwise widely occurring genes while at the same time eliminating genes located in regions of difference and in genomic islands, which make up 21% of the *B. cenocepacia* J2315 genome (6). The output was a comprehensive list of 5,702 possible interactions present in at least 50% of the input strains, ranked by interaction *P* value. Only the 100 highest ranking interactions with the smallest *P* values were further analyzed because benchmark data sets showed that true interactions can usually be found among the top 100 ranked ones (44). Limited accessibility within the ncS35 secondary structure meant that the predicted interactions are generally rather short, on average, only 10 nt long. Moreover, the accessible sequences have a high GC content that is equal to the background. Because of these properties, most interactions of ncS35 with putative target genes are assigned a high *P* value and therefore a uniformly high false-discovery rate because of the high likelihood that the interaction sequence is present in the putative target by pure chance. The ranking of a certain interaction, therefore, depended rather heavily on the calculated phylogenetic tree because the *P* values used for ranking of results are combined from all conserved interactions, weighted on the basis of phylogenetic distance. Phylogenetic trees can vary; therefore, the analysis was repeated five times and only interactions occurring among the 100 highest ranking ones on all five lists of results were retained. Predicted interactions that fell outside the 5′ UTRs of putative targets were discarded.

### Statistics.

Each experiment included at least three biological replicates, and representative images were taken. Results were checked for normality. For normally distributed data, an independent-sample *t* test or ANOVA was performed; data not normally distributed were analyzed with a Mann-Whitney or a Kruskal-Wallis test. Differences were considered significant when the *P* value was <0.05.

### Accession number(s).

The raw RNA-seq data obtained in this study were submitted to ArrayExpress under accession number E-MTAB-5526.
