# Supplementary figures and images for: The Small RNA ncS35 Regulates Growth in Burkholderia cenocepacia J2315
Source: mSphere. 2018 Jan 10;3(1):e00579-17. doi: 10.1128/mSphere.00579-17 (PMC5760752; doi:10.1128/mSphere.00579-17)

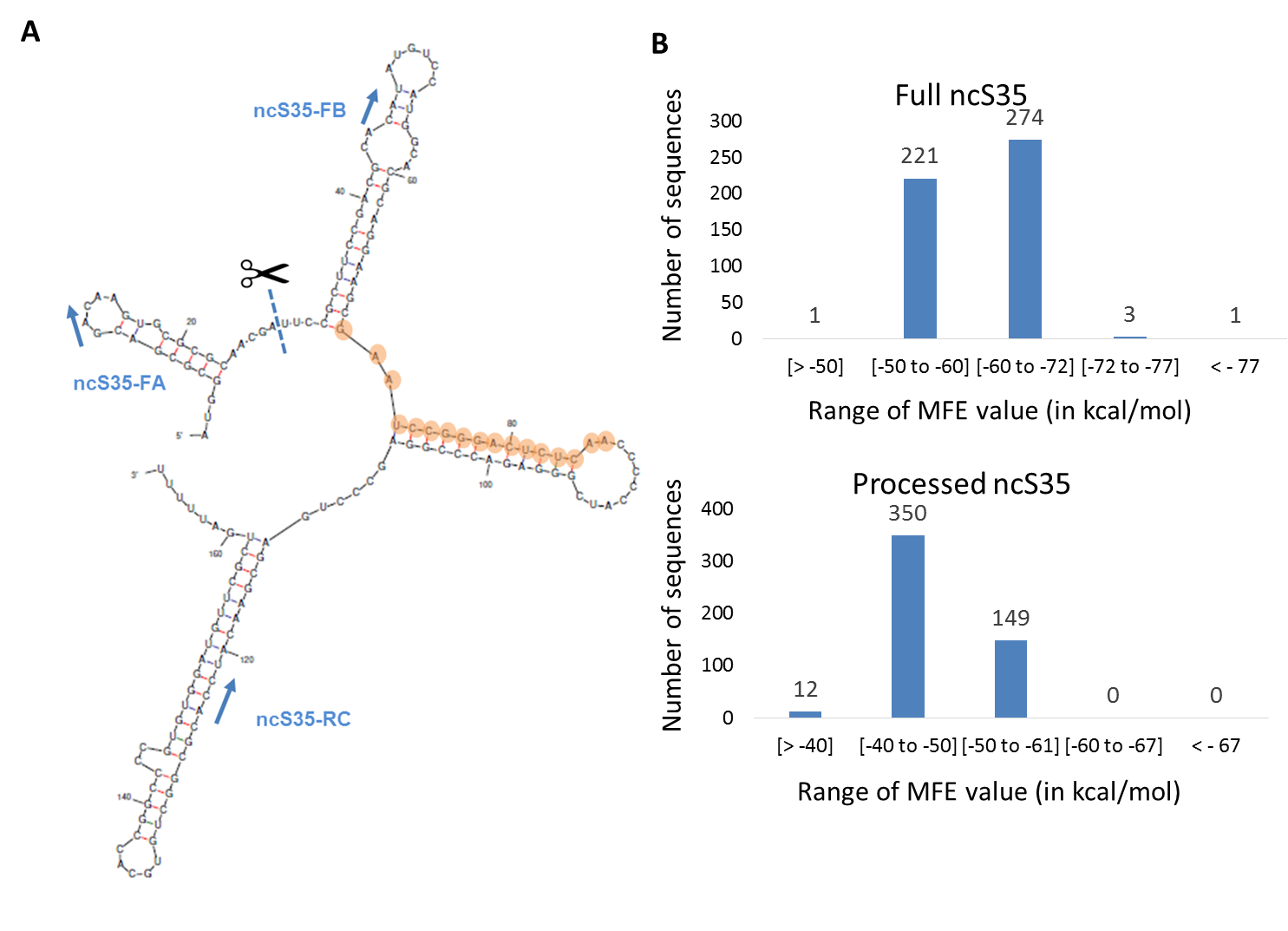


**Processed ncS35**

**Full length ncS35**

**Figure S1**

Supplement: FIG S1 [file sph001182448sf1.docx]

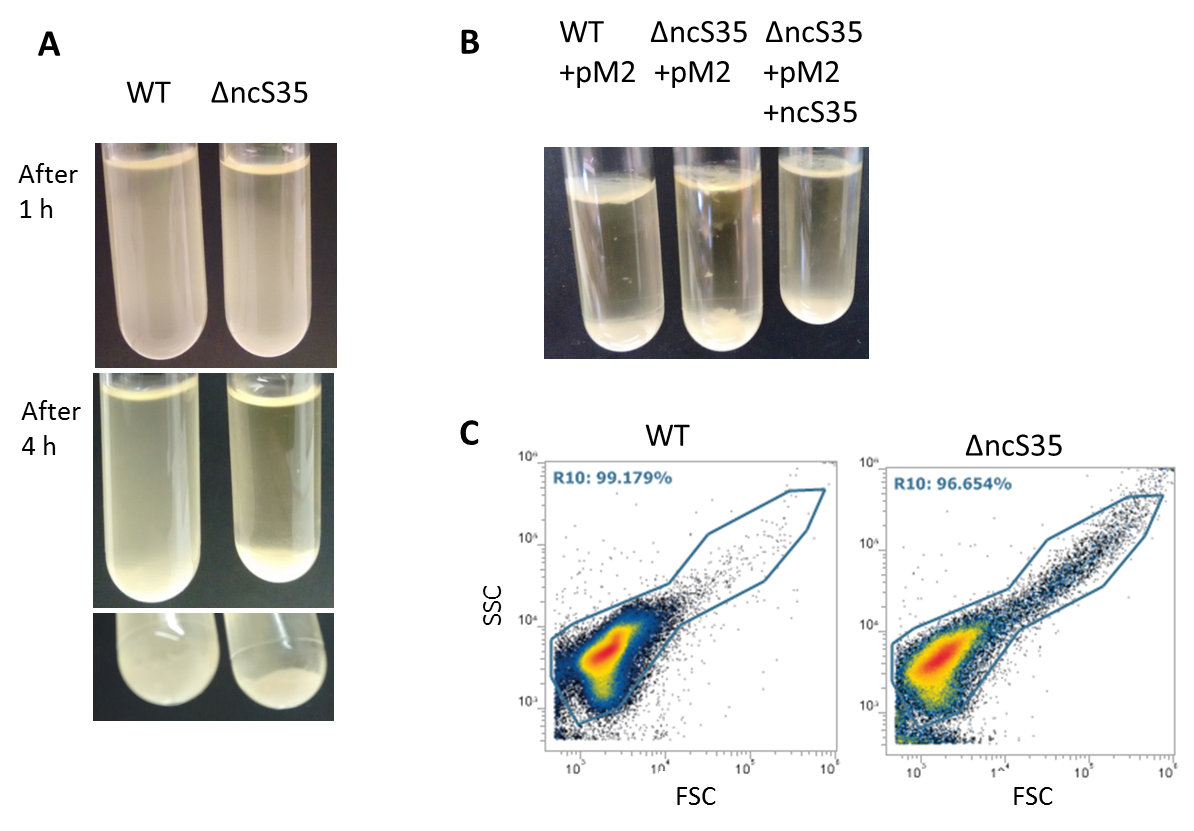


**Figure S2**

Supplement: FIG S2 [file sph001182448sf2.docx]

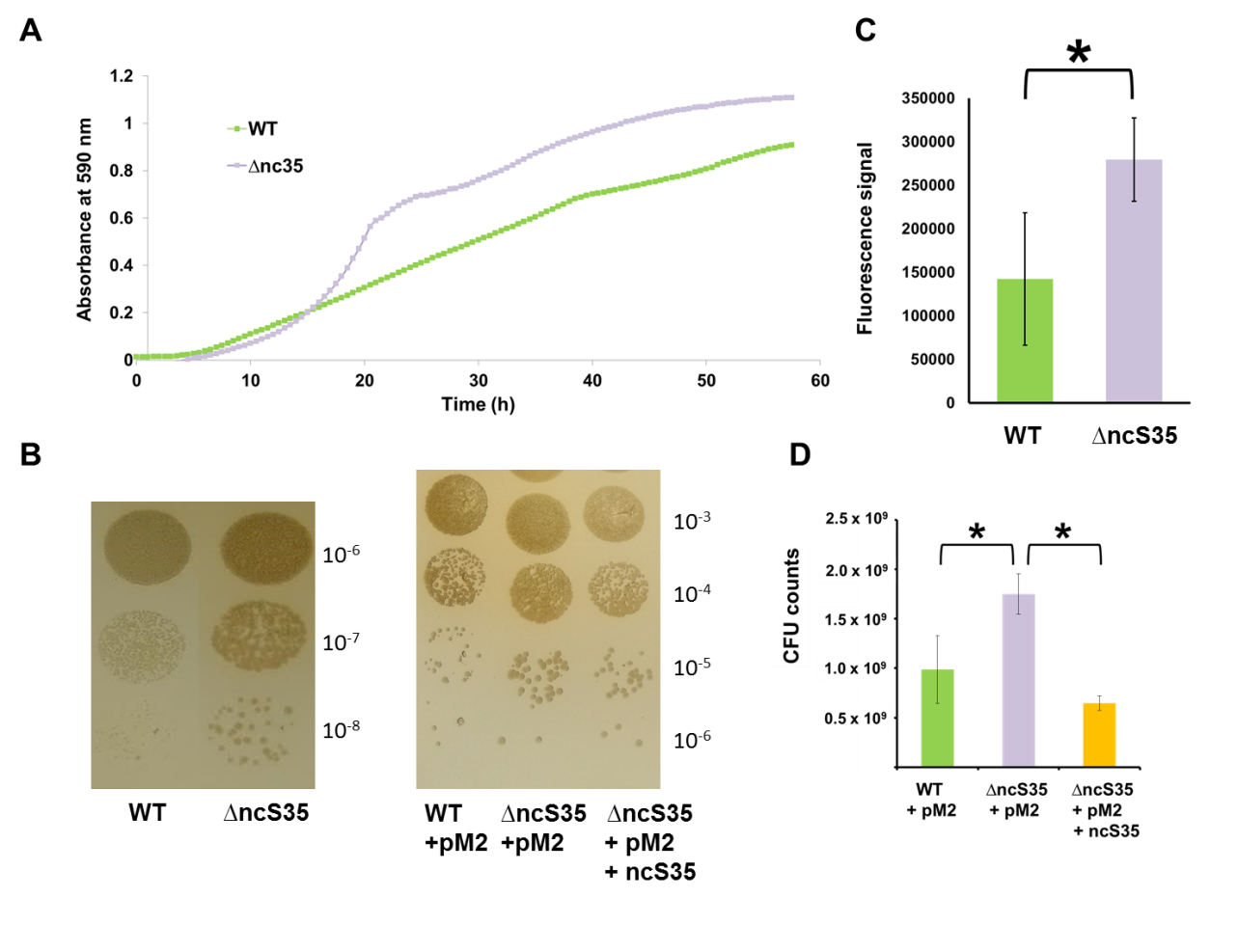


**Figure S3**

Supplement: FIG S3 [file sph001182448sf3.docx]

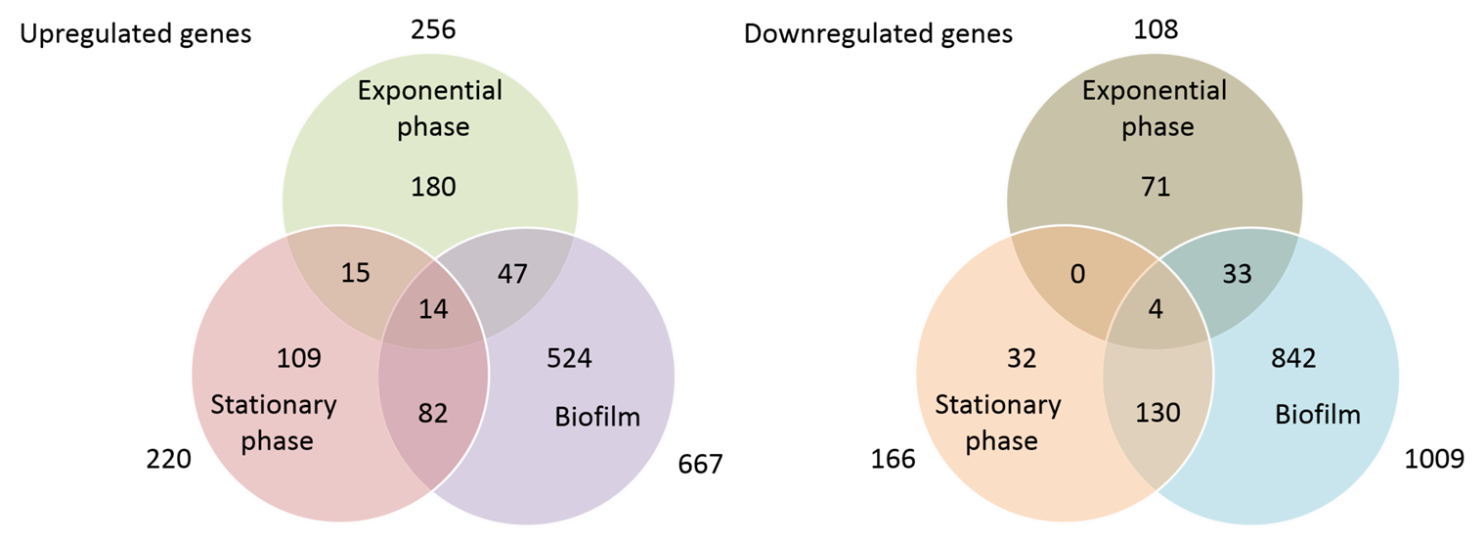


**Figure S5**

Supplement: FIG S5 [file sph001182448sf5.docx]

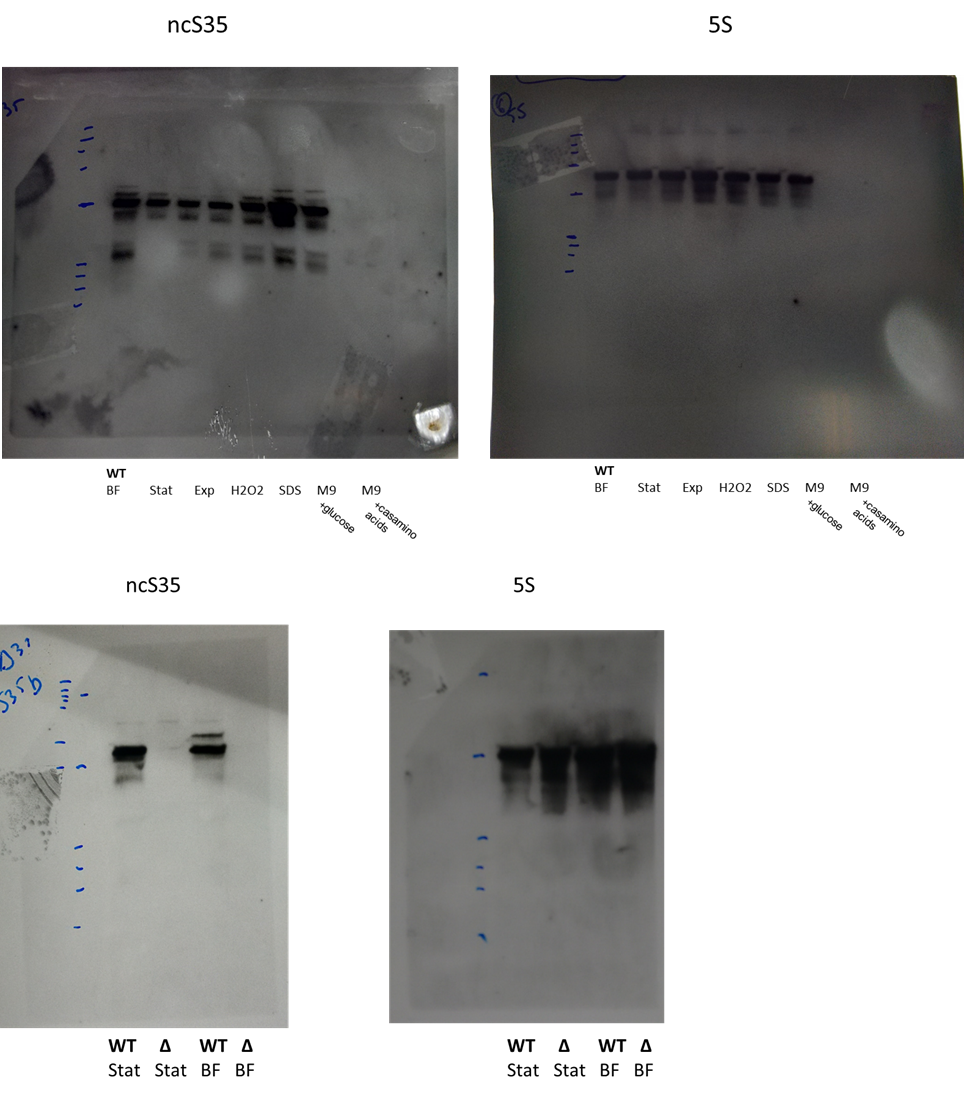


**Figure S6**

Supplement: FIG S6 [file sph001182448sf6.docx]
